# *Clonorchis sinensis* infection alters the methylation and hydroxymethylation of hepatocellular carcinoma

**DOI:** 10.3389/fcimb.2026.1799003

**Published:** 2026-07-03

**Authors:** Caibiao Wei, Yuanbo Li, Lingling Zhou, Yulong Xu, Junxian Chen, Min Fang, Taijun Huang, Jie Gao, Fengfei Liu, Jingyu Su, Jian-Kang Zhu, Weilong Yang

**Affiliations:** 1 Department of Clinical Laboratory, Guangxi Medical University Cancer Hospital, Nanning, China; 2 Department of Blood Transfusion, Guangxi Medical University Cancer Hospital, Nanning, China; 3 Department of Clinical Laboratory, The First Affiliated Hospital of Guangxi Medical University, Nanning, China; 4 Genetic Metabolism Center Laboratory, Guangxi Zhuang Autonomous Region Maternal and Child Health Care Hospital, Nanning, China; 5 Institute of Advanced Biotechnology, Institute of Homeostatic Medicine, and School of Medicine, Southern University of Science and Technology, Shenzhen, China; 6 Guangzhou Women and Children's Medical Center, Guangzhou Medical University, Guangzhou, China

**Keywords:** *Clonorchis sinensis*, hepatocellular carcinoma, hydroxymethylation, methylation, prognosis, whole-genome bisulfite sequencing

## Abstract

**Background:**

The molecular mechanisms contributing to the poor prognosis of *Clonorchis sinensis* (*Cs*)-infected hepatocellular carcinoma (HCC) remain poorly understood, especially when it comes to DNA methylation.

**Methods:**

Using parallel whole-genome bisulfite sequencing (WGBS) and whole-genome oxidative bisulfite sequencing (oxWGBS), we characterized the profiles of 5-methylcytosine (5mC) and 5-hydroxymethylcytosine (5hmC) in *Cs*-infected HCC tumors and adjacent non-tumor tissues at single-nucleotide resolution. These profiles were compared with data from non-*Cs*-infected HCC available in the GEO database. Additionally, we investigated the impact of DNA methylation modifications on differential gene expression and prognosis in *Cs*-infected HCC by analyzing HCC related data from the TCGA database and RNA-seq data from previous studies.

**Results:**

Here, we reported the methylation and hydroxymethylation landscapes of *Cs*-infected and *Cs*-non-infected HCC patients at single-nucleotide resolution by WGBS and oxWGBS, respectively. 29 differentially methylated regions (DMRs) and 13 differentially hydroxymethylated regions (DhMRs) were found in *Cs*-infected HCC tumor tissues compared to *Cs*-non-infected HCC tumor tissues. Following, we identified 28 differentially methylated/hydroxymethylated-associated genes (DAGs/DhAGs), two genes (DHDH and KCNQ3) of which were significantly correlated with the overall survival of HCC. Finally, we also revealed that four Cytosine-phosphate-Guanine sites in the promoter of DAGs/DhAGs certainly affect the survival outcomes of HCC.

**Conclusions:**

This study provides the first comprehensive characterization of DNA methylome and hydroxymethylome landscapes in *Cs*-infected HCC, shedding light on the impact of on HCC methylation and offering new insights into the role of *Cs* in HCC progression. Our findings also highlight the key DMRs/DhMRs and DAGs/DhAGs in *Cs*-infected HCC tumors, which could serve as promising epigenetic biomarkers for predicting the prognosis and therapeutic targets of *Cs*-associated HCC.

## 1 Introduction

Hepatocellular carcinoma (HCC) is the predominant form of liver cancer, with an incidence rate ranking 6th globally and a mortality rate placing it as the 3rd leading cause of cancer-related deaths worldwide (Bray et al., 2024). HCC development is influenced by a complex interplay of genetic mutations, environmental exposures, chronic infection (e.g., hepatitis viruses and liver fluke), excessive alcohol consumption, and non-alcoholic fatty liver disease (NAFLD). Among these liver fluke, *Clonorchis sinensis* (*Cs*) infection has been classified as a Class I carcinogen by the International Agency for Research on Cancer, and increasing evidence supports that its infection is associated with poor prognosis in HCC (Qian et al., 2016; Li et al., 2023; Liu et al., 2023; Lin et al., 2024; Ni et al., 2024).

*Cs* is a foodborne parasitic infection transmitted to humans through the consumption of raw or undercooked freshwater fish containing infective *Cs* metacercariae. The parasite itself, along with its secretory and excretory products (*Cs*ESP), can induce pathological changes such as bile duct dilatation, cholangiocyte proliferation, and thickening of the ductal walls, which may progress to conditions like cholangitis, cholecystitis, liver fibrosis, and ultimately cholangiocarcinoma and HCC (Qian et al., 2012; Qian et al., 2024). *In vivo* studies have demonstrated that *Cs* infection enhances liver cancer risk in mice by stimulating the proliferation of hepatic progenitor cells (Qi et al., 2022), while *in vitro* studies show that its particulate protein drives hepatocyte malignant transformation by activating the RAS/MAPK/ERK and PI3K/AKT signaling pathways via epidermal growth factor receptors (Wang et al., 2021). Furthermore, our previous studies have confirmed that *Cs* infection significantly amplifies HCC stemness and promotes angiogenesis, thereby accelerating the malignant progression of HCC (Lin et al., 2024; Wei et al., 2024). Totally, current evidence suggests that *Cs* infection induces phenotypic changes through multiple mechanisms, which contribute to the progression of HCC.

The development and progression of HCC involve a series of genetic and epigenetic alterations, including genomic mutations, DNA methylation, histone modifications, and chromatin remodeling (Li et al., 2018). Among these, DNA methylation is a key and widespread epigenetic mechanism regulating tumor development (Gao et al., 2014; Manzo et al., 2017). It does not alter the genetic code but instead affects the readability of DNA, thereby regulating gene transcription and frequently leading to gene silencing (Ye and Li, 2014; Nishiyama and Nakanishi, 2021). 5mC, formed by methylation at the 5′ position of cytosine, is the predominant DNA methylation mark in mammalian genomes (Pfeifer et al., 2013). It is predominantly found at Cytosine-phosphate-Guanine (CpG) sites, where hypermethylation of CpG islands is typically associated with transcriptional repression, while hypomethylation can lead to aberrant gene activation (López et al., 2017). Such aberrant methylation events play crucial roles in HCC progression (Gao et al., 2014; Ye et al., 2016). The oxidized derivative 5-hydroxymethylcytosine (5hmC), generated by the catalytic action of Ten-Eleven Translocation (TET) enzymes, has been shown to undergo a global reduction and redistribution across multiple solid tumors (Liu et al., 2013). It functions as both an essential intermediate in active DNA demethylation and an independent epigenetic mark with unique regulatory roles (Pfeifer et al., 2013). Previous studies have demonstrated that 5hmC levels in HCC tissues are significantly lower than those in adjacent non-tumorous liver tissues, and correlate strongly with poor differentiation and microvascular invasion (Chen et al., 2017). Moreover, loss of 5hmC has been reported to induce chemoresistance in HCC through the 5hmC/PCAF/AKT axis (Guo et al., 2023). Therefore, alterations in 5mC and 5hmC are crucial to understanding the molecular mechanisms underlying HCC initiation and progression.

Accumulating evidence indicates that host-parasite interactions can reshape host DNA (hydroxy)methylation patterns (Mei et al., 2019; Hammam et al., 2020; Sagonas et al., 2020). Parasitic infections may modulate host methylation status during invasion or via parasite-derived extracellular vesicles, which in turn alter gene expression and promote oncogenic processes (Mei et al., 2019; Vega-Benedetti et al., 2022). However, to date, no studies have reported the comprehensive 5mC and 5hmC methylation landscapes of HCC in the context of *Cs* infection. In this study, we characterized the 5mC and 5hmC modification patterns in *Cs* associated HCC using parallel whole-genome bisulfite sequencing (WGBS) and whole-genome oxidative bisulfite sequencing (oxWGBS). These patterns were compared with those of non-*Cs*-infected HCCs from public databases to identify distinct methylation and hydroxymethylation profiles associated with *Cs* infection. Through this approach, we aimed to better understand the underlying reasons for the poor prognosis of *Cs*-infected HCC, identify potential biomarkers for its prediction, and uncover new possibilities for personalized treatment strategies for liver cancer patients.

## 2 Materials and methods

### 2.1 Samples collection

Tissue samples were collected from four *Cs*-infected, treatment-naive HCC patients who underwent surgical resection at the Department of Hepatobiliary Surgery, Guangxi Medical University Cancer Hospital (Nanning, China). Patients were classified into two groups based on *Cs* infection status: *Cs*-infected hepatocellular carcinoma (*Cs*^+^ HCC) patients and non-infected hepatocellular carcinoma (*Cs*^-^ HCC) patients. *Cs* infection was diagnosed based on imaging, pathology, or fecal tests detecting *Cs* eggs or adult worms (**Supplementary Figure 1A**). For each patient, paired tumor (T) and adjacent non-tumor (P) liver tissues were collected. Tumor tissues, with characteristic macroscopic features, were obtained from tumor nodules and confirmed by histological examination (H&E staining) (**Supplementary Figure 1B**). Adjacent non-tumor tissues, free of detectable tumor cells, were collected from areas 5 cm from the tumor margin. Accordingly, four types of samples were included in this study: *Cs*^+^ HCC tumor tissues (*Cs*^+^_T), *Cs*^+^ HCC adjacent non-tumor tissues (*Cs*^+^_P), *Cs*^-^ HCC tumor tissues (*Cs*^-^_T), and *Cs*^-^ HCC adjacent non-tumor tissues (*Cs*^-^_P). The study was approved by the Guangxi Medical University Cancer Hospital Ethical Review Committee (LW2024143) and conducted in accordance with the Declaration of Helsinki. Upon admission, all patients provided written consent for the analysis and publication of their anonymized medical data for research purposes. The clinical characteristics of all enrolled patients, including age, sex, serum AFP level, HBV infection status, tumor size, BCLC stage, tumor number, microvascular invasion (MVI) status, liver cirrhosis, Edmondson grade, tumor satellite, and microvessel density (MVD), are summarized in **Supplementary Table 1**.

### 2.2 Bisulfite sequencing and oxidative bisulfite sequencing library preparation and sequencing

After quality control, lambda DNA was added as a bisulfite conversion efficiency control. Genomic DNA was fragmented to 200–400 bp using a Covaris M220 Focused-ultrasonicator (Covaris, USA). The fragmented DNA was then end-repaired, 3’ adenylated, and ligated with methylation-modified sequencing adapters. Next, 5hmC was oxidized to 5fC, followed by bisulfite treatment using the EZ DNA Methylation Gold Kit (Zymo Research). Unmethylated C and oxidized 5fC were converted to U (T after PCR amplification), while methylated C remained unchanged. PCR amplification was performed to generate the final oxWGBS library. After confirming library quality, a balanced library was added for sequencing on the Illumina HiSeq 3000 using a 150 bp paired-end model. Sequencing was performed using the Sequencing by Synthesis (SBS) method, where fluorescently labeled dNTPs were incorporated, and fluorescence signals were captured to generate sequence data.

### 2.3 BS-seq and oxBS-seq data processing

Firstly, Trim Galore (v.0.6.10) (Zhao et al., 2023) was used to remove low-quality reads and adapters from raw sequencing reads and obtain clean data. Next, clean data were aligned to the hg38 reference genome using BSMAP (v.2.90) (Nunn et al., 2021) with the following options: -p 10 -v 0.05. The PCR duplicates were removed by sambamba (v.0.6.6) (Tarasov et al., 2015). methratio.py (a Python-based script embedded in BSMAP) was used to extract the methylation measurements for each CpG site from aligned reads. Mapped CpG sites with coverage no less than 10-fold were included for further analysis.

Metilene (v 0.2.8) was performed to analyze groupwise differentially methylated regions (DMRs) and differentially hydroxymethylated regions (DhMRs) with the following parameters: |Δβ| > 0.2, number of CpG > 10 per region, and FDR < 0.05.

### 2.4 Distribution analysis of 5mC and 5hmC

Genomic annotations of the hg38 were obtained from Ensembl (https://asia.ensembl.org/index.html). Genomic regions annotated as 5′-untranslated region (5′-UTR), exon, intron, intergenic, 3′-untranslated region (3′-UTR), gene body and transposable elements (TEs). Next, the bedtools (v2.30.0) (Li et al., 2022) was used to identify the CpG sites, DMRs and DhMRs in 5′-UTR, exon, intron, intergenic, and 3′-UTR. The average 5mC and 5hmC levels of genome were plotted by R packages ‘CMplot’ (v.4.5.1) and the length of each bin was 100 Kb. The average 5mC and 5hmC levels of gene body and TEs were calculated by computeMatrix in deeptools (v.3.5.1) and divided into 50 bins. Finally, Circos (v0.69-8, https://github.com/vigsterkr/circos) was used to display the distribution of DMRs and DhMRs with 200 Kb windows.

### 2.5 Gene enrichment analysis

We used the Metascape (https://metascape.org/gp/index.html#/main/step1) to do gene enrichment analysis with default parameters.

### 2.6 TCGA database analysis

The R packages ‘TCGAplot’ (v.7.0.1) was used to do analysis and TCGA-LIHC cohort was performed to analysis about HCC. Specifically, gene_methylation_scatter was employed to assess the correlation between gene expression and promoter methylation, using 5mC levels within −2 kb to +500 bp of the transcription start site and corresponding mRNA expression values derived from RNA-seq data. Additionally, methy_kmplot was used to perform survival analysis based on the promoter methylation of specific genes, and tcga_kmplot was utilized to assess survival outcomes associated with the expression of individual genes.

### 2.7 Integrative analysis of RNA−seq data

The gene expression matrix of *Cs*^+^_T and *Cs*^-^_T was obtained from our previous study (GEO: GSE276855). R package ‘DEseq2’ (v.1.44.0) to normalize gene expression and detect differentially expressed genes (|Fold Change| > 2 and *p* < 0.05). For the analysis of DNA methylation-related genes, we specifically focused on well-established regulators of DNA methylation and demethylation, which were selected based on literature evidence as key enzymes responsible for 5mC and 5hmC regulation (Chen and Zhang, 2020; Chen et al., 2022).

### 2.8 Statistical analysis

R software (v.4.3.0) was used for statistical analysis. Principal component analysis (PCA) was done by prcomp function. Additional details regarding the analytical methods, parameters, and example R code have been made publicly available on GitHub for reproducibility: https://github.com/506803062/HCC_methylation.

### 2.9 Collection and preparation of Cs-produced excretory/secretory products (CsESPs)

Adult *Cs* worms were cultured *in vitro*, and the culture medium was collected every 6–12 h over 48 h. Media from the first 24 h and the last 24 h were pooled separately and stored at 4 °C. The collected supernatants were clarified by centrifugation at 12,000 rpm for 30 min at 4 °C. The supernatant was then dialyzed against PBS and concentrated by sucrose cushion ultracentrifugation or lyophilization depending on downstream applications. Protein concentration and molecular characteristics were determined. Preparations were aliquoted and stored at −80 °C. Prior to use, samples were sterilized through a 0.22-μm filter.

### 2.10 Cell culture

The Type Culture Collection of the Type Culture Collection of the Chinese Academy of Sciences (Shanghai, China) was the source of the human hepatocellular carcinoma cell lines MHCC97H and Huh7. DMEM supplemented with 10% fetal bovine serum (Wisent, Canada) was used to culture the cells at 37 °C in a humidified atmosphere containing 5% CO_2_. The cells were further treated with 50 μg/mL CsESPs or an equal volume of PBS for 48 hours.

### 2.11 Reverse Transcription-quantitative Polymerase Chain Reaction (RT-qPCR)

Total RNA was extracted from MHCC97H and Huh7 cells treated with CsESP or PBS using TRIzol reagent (Invitrogen, USA). cDNA was synthesized from 1.0 μg of RNA using a Reverse Transcription Master Kit (Takara, Japan).RT-qPCR was performed on a qTOWER3 Real-Time PCR System (Jena, Germany) using TB Green Premix Ex Taq II Fast (2×) (Takara, Japan) under standard cycling conditions (95 °C for 30 s, followed by 39 cycles of 95 °C for 5 s and 60 °C for 30 s). Gene expression levels were normalized to GAPDH and calculated using the 2^−ΔΔCt method. All reactions were performed in triplicate. Primers for DHDH and KCNQ3 were designed and synthesized by Sangon Biotech (Shanghai, China).

## 3 Results

### 3.1 Methylation and hydroxymethylation landscapes of *Cs*^+^ and *Cs*^-^ HCC patients

To characterize the methylation atlas of *Cs*-associated HCC and ensure a balanced comparison with the publicly available dataset of *Cs*^-^ HCC patients, we performed a comparative analysis of 5mC and 5hmC modification patterns in tumor and adjacent non-tumor tissues from both *Cs*^+^ HCC and *Cs*^-^ HCC patients using WGBS and oxWGBS. The sequence data of 4 pairs of *Cs^+^*_T and *Cs^+^*_P, was produced in this study, and the detailed patient information was provided in **Supplementary Table 1**. Additionally, the sequence data of 4 pairs of *Cs^-^*_P and *Cs^-^*_T from *Cs*^-^ HCC patients was downloaded from publicly database (GEO: GSE70091), serving as a control group for comparative analysis. After quality control, each sample had approximately 354.54 million clean reads, with WGBS libraries sequenced to an average depth of 34.13-fold and mapping rates exceeding 82.26%, and oxWGBS libraries sequenced to an average depth of 36.93-fold with mapping rates exceeding 83.81% (**Supplementary Table 2**). In total, 1.01 to 7.80 million CpG sites were identified with a cutoff criterion of at least 10-fold coverage, enabling the construction of comprehensive genome-wide single-base-resolution maps of 5mC and 5hmC modifications in *Cs^+^* HCC and *Cs^-^* HCC patients (**Supplementary Table 3**).

**Figure 1:**
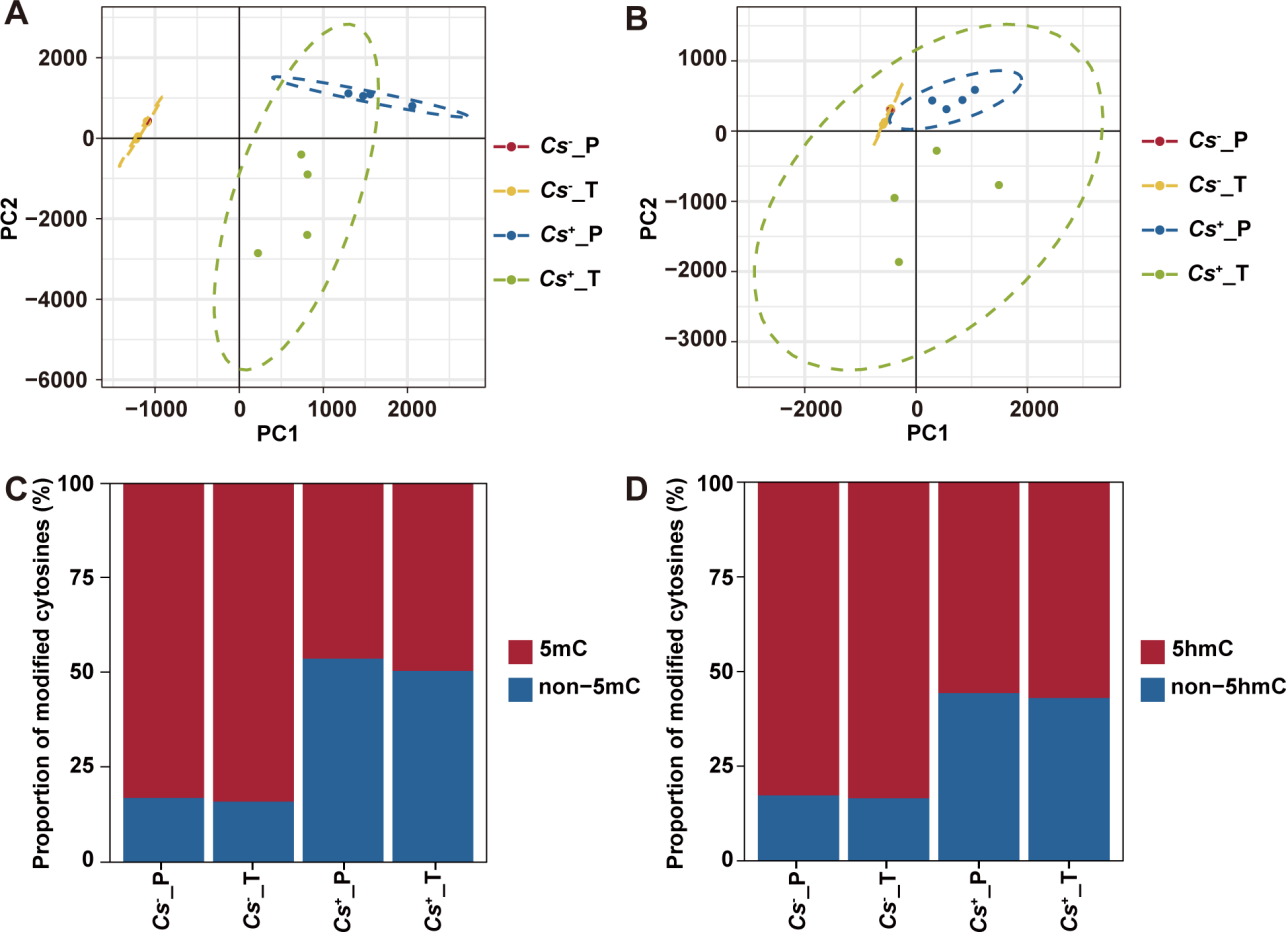
Profile of DNA methylation and hydroxymethylation in *Cs*^+^ and *Cs*^-^ HCC patients. A, **(B)** The PCA plot highlights distinct DNA methylation patterns **(A)** and hydroxymethylation patterns **(B)** in *Cs^-^*_T, *Cs^-^*_P, *Cs*^+^_T and *Cs*^+^_P groups. C, **(D)** Staking bar plot shows the proportion of 5mC **(C)** and 5hmC **(D)** at CpG sites in *Cs^-^*_T, *Cs^-^*_P, *Cs*^+^_T and *Cs*^+^_P groups.

Principal component analysis (PCA) of the 5mC data revealed clear segregation between *Cs^+^* HCC and *Cs^-^* HCC groups, underscoring distinct methylation profiles associated with *Cs* infection (**Figure 1A**). While partial overlap was observed between *Cs^+^*_T and *Cs^+^*_P, the separation was still evident, suggesting significant 5mC alterations between tumor and adjacent tissues. Similarly, PCA of 5hmC data showed partial segregation between *Cs^+^* HCC and *Cs^-^* HCC groups, reflecting infection-specific methylation remodeling (**Figure 1B**). Significantly, the proportion of CpG sites bearing a certain degree of 5mC was highest in *Cs^-^*_T (84.01%), followed by *Cs^-^*_P (83.04%), *Cs^+^*_T (49.52%), and *Cs^+^*_P (46.27%) (**Figure 1C**). Similarly, 5hmC levels showed a comparable pattern, with proportions of 5hmC at 83.49%, 82.71%, 57.02%, and 55.74% in *Cs^-^*_T, *Cs^-^*_P, *Cs^+^*_T and *Cs^+^*_P, respectively (**Figure 1D**). Moreover, the proportions of 5mC and 5hmC at CpG sites in each sample was shown in **Supplementary Figure 2**.

### 3.2 Distribution of 5mC and 5hmC across genomic regions

**Figure 2:**
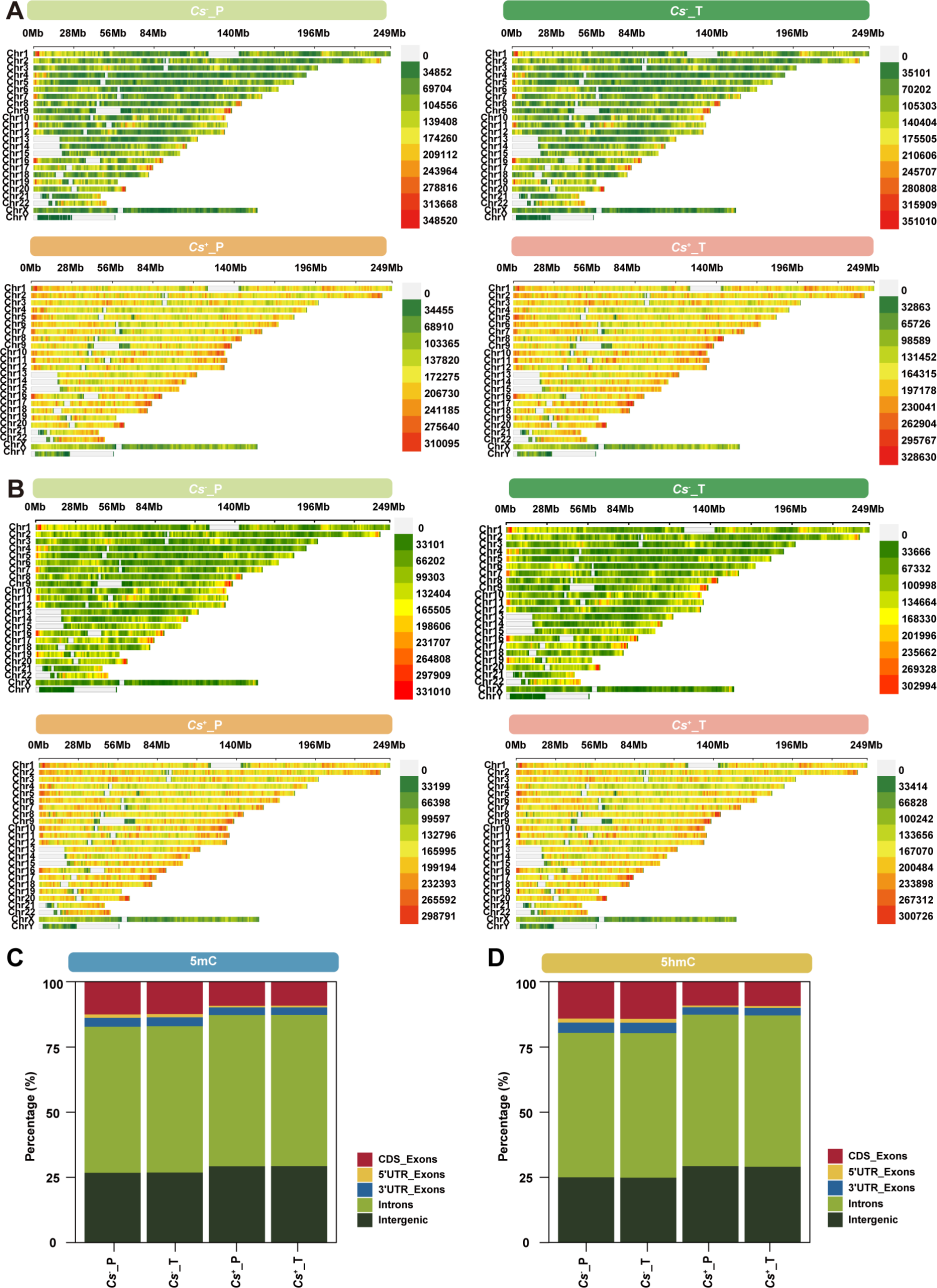
Genomic distribution of 5mC and 5hmC in *Cs*^+^ and *Cs*^-^ HCC patients. A, **(B)** The density distribution of methylation **(A)** and hydroxymethylation **(B)** sites on chromosomes in *Cs^-^*_T, *Cs^-^*_P, *Cs*^+^_T and *Cs*^+^_P groups. C, **(D)** Distribution of 5mC **(C)** and 5hmC **(D)** across functional genomic regions in *Cs^-^*_T, *Cs^-^*_P, *Cs*^+^_T and *Cs*^+^_P groups.

To investigate the genomic features of 5mC and 5hmC in *Cs*^+^ HCC and *Cs*^-^ HCC patients, we analyzed their chromosomal and regional distribution across different genomic elements. Firstly, the density distribution of methylation and hydroxymethylation sites on chromosomes in *Cs*^+^ HCC and *Cs*^-^ HCC patients were displayed, and it was not uniform (**Figures 2A, B**). Furthermore, the 5mC and 5hmC distribution across genomic features was also presented (**Figures 2C, D**). In all groups, 5mC and 5hmC were predominantly enriched in introns and intergenic regions. Finally, the genomic features of 5mC and 5hmC in each sample were also shown in **Supplementary Figures 3, 4**.

**Figure 3:**
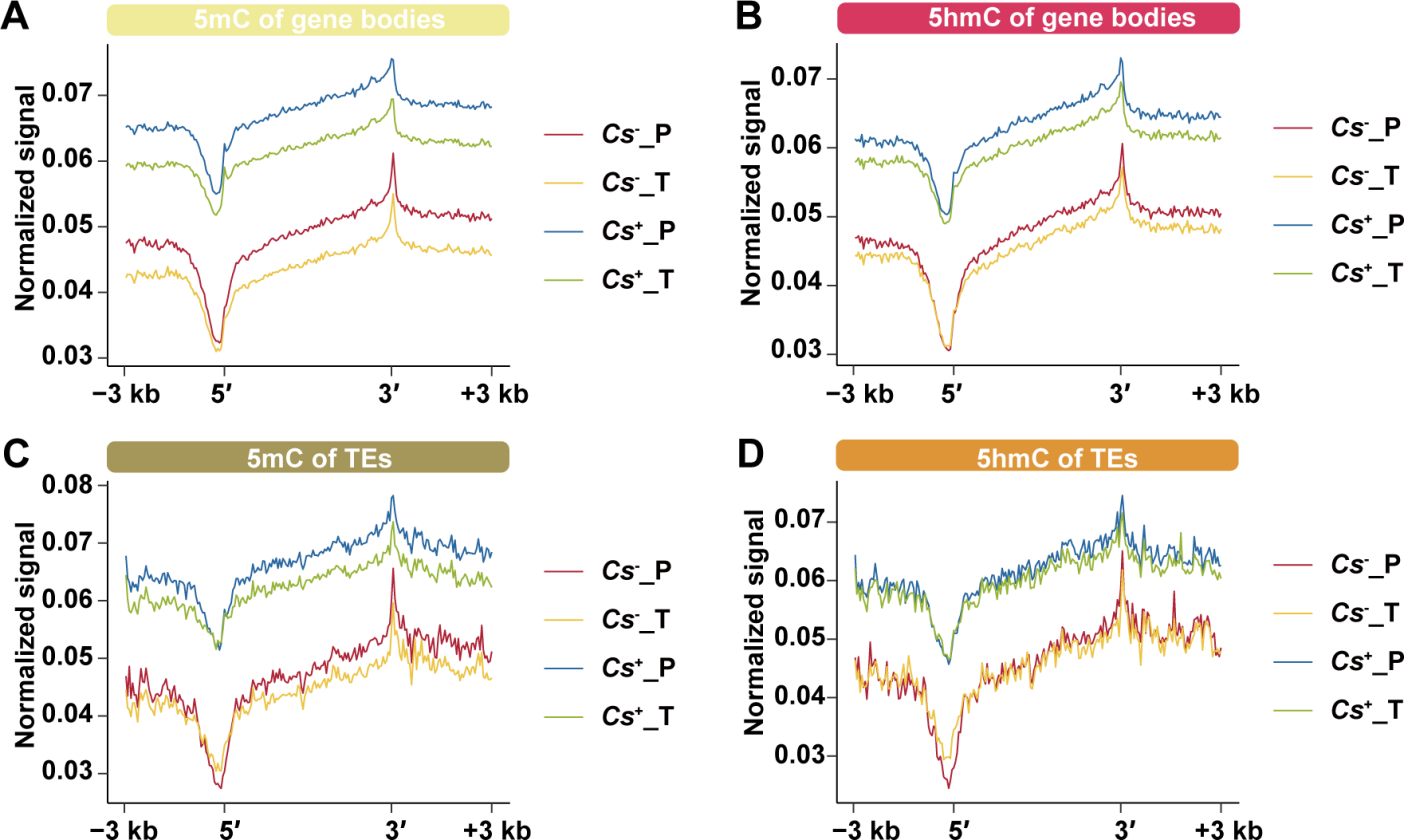
DNA methylation and hydroxymethylation levels of gene bodies and TEs in *Cs*^+^ and *Cs*^-^ HCC patients. A, **(B)** Average DNA methylation **(A)** and hydroxymethylation **(B)** levels of gene bodies in *Cs^-^*_T, *Cs^-^*_P, *Cs*^+^_T and *Cs*^+^_P groups. C, **(D)** Average DNA methylation **(C)** and hydroxymethylation **(D)** levels of TEs in *Cs^-^*_T, *Cs^-^*_P, *Cs*^+^_T and *Cs*^+^_P groups.

Next, we analyzed the average 5mC and 5hmC levels of gene bodies and TEs in *Cs*^+^ HCC and *Cs*^-^ HCC patients. For gene bodies, both average 5mC and 5hmC levels exhibited a similar distribution pattern across the four different groups, with lower levels near the transcription start sites (TSS) and higher levels near the transcription termination sites (TTS) (**Figures 3A, B**). Notably, the overall levels of both 5mC and 5hmC in gene bodies were significantly higher in *Cs*^+^ HCC compared to *Cs*^-^ HCC patients. Within each group, tumor tissues exhibited significantly lower 5mC levels of gene bodies compared to adjacent normal tissues (**Figure 3A**), which was also observed for 5hmC (**Figure 3B**). For TEs, the average levels of both 5mC and 5hmC were similar to that in gene bodies, but the difference between tumor and adjacent normal tissues was smaller (**Figures 3C, D**). In detail, the profiles of DNA methylation and hydroxymethylation levels of gene bodies and TEs in each sample were presented in **Supplementary Figure 5**.

### 3.3 Characterization of methylation patterns in *Cs*^+^ HCC and *Cs*^-^ HCC patients

**Figure 4:**
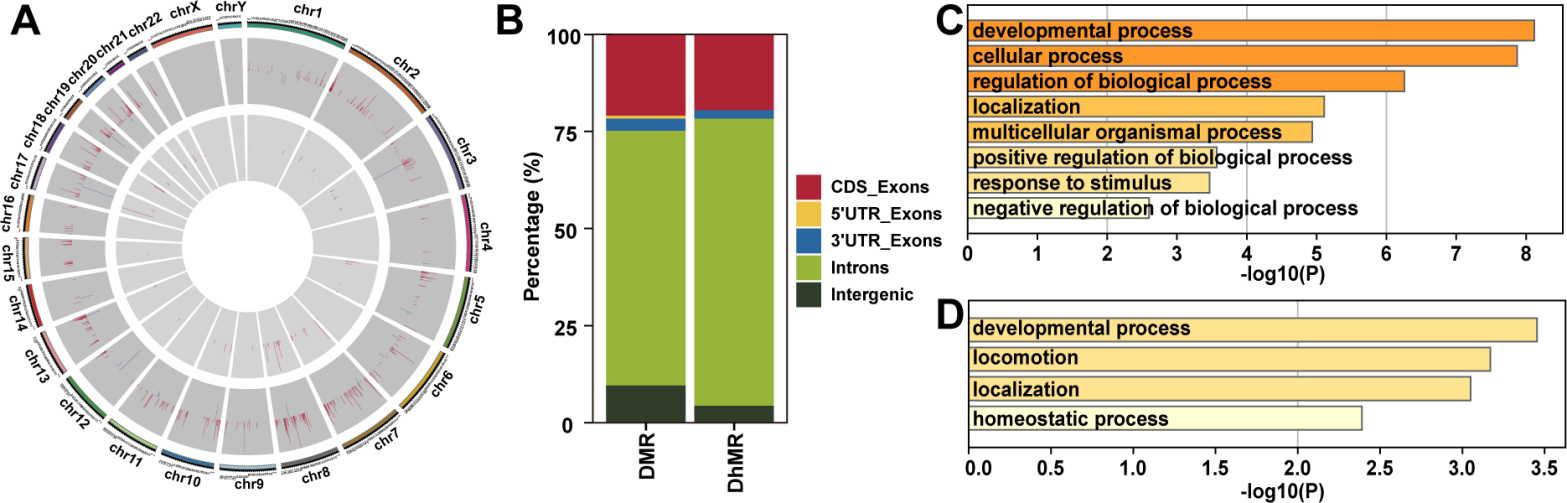
Identification of DMRs and DhMRs between *Cs*^+^ HCC tumors and adjacent non-tumor tissues. **(A)** The Circos plot illustrates the genomic distribution of DMRs and DhMRs between *Cs*^+^ HCC tumors and adjacent non-tumor tissues across all chromosomes. Each individual bar in the outer and inner circles represents a DMR or DhMR, respectively. The height of each bar corresponds to the extent of methylation difference (Δβ value), with red bars indicating hypermethylation and blue bars indicating hypomethylation. **(B)** Distribution of DMRs and DhMRs between *Cs*^+^ HCC tumors and their adjacent non-tumor tissues across genomic regions. C, **(D)** GO analysis of DAGs/DhAGs based on DMRs **(C)** and DhMRs **(D)**.

Next, we identified DMRs and DhMRs, which consist of multiple consecutive CpG sites, between the *Cs^+^*_T and *Cs^+^*_P groups. A total of 725 DMRs and 65 DhMRs were identified by comparing the 5mC and 5hmC profiles between the two groups. Within these DMRs and DhMRs, 695 were hypermethylated and 30 were hypomethylated (**Supplementary Table 4**); 64 were hyperhydroxymethylated and 1 were hypohydroxymethylated (**Supplementary Table 5**). What’s more, the distribution of all DMRs and DhMRs were shown in **Figure 4A**. Genomic region analysis revealed that DMRs and DhMRs were predominantly located in introns and CDS exons, while they were less frequently observed in intergenic regions and untranslated regions (UTRs) (**Figure 4B**). To explore the functional implications of these methylation and hydroxymethylation changes, gene ontology (GO) enrichment analysis was conducted for DMR/DhMR-associated genes (DAGs/DhAGs) between the *Cs^+^*_T and *Cs^+^*_P groups. DAGs were significantly enriched in pathways related to development, cellular functions, and biological regulation, all of which were linked to cancer-related biological pathways (**Figure 4C**). Similarly, DhAGs were also associated with pathways related to cancer progression, including development, locomotion, and localization (**Figure 4D**).

Additionally, a similar analysis was performed for *Cs*^-^_T and *Cs*^-^_P groups, and a total of 559 DMRs and 539 DhMRs were identified. Within these DMRs and DhMRs, 173 were hypermethylated and 386 were hypo-methylated (**Supplementary Table 6**); 131 were hyperhydroxymethylated and 408 were hypo-hydroxymethylated (**Supplementary Table 7**). The distribution of these DMRs and DhMRs was also presented (**Supplementary Figure 6A**). Otherwise, these DMRs and DhMRs were found across various regions, with the highest proportion observed in CDS exons (**Supplementary Figure 6B**). GO analysis revealed that the associated genes were primarily involved in developmental processes and biological regulation, supporting their functional relevance in tissue homeostasis and tumor biology (**Supplementary Figures 6C, D**).

### 3.4 Characterization of methylation dynamics between *Cs*^+^ HCC and *Cs*^-^ HCC patients

**Figure 5:**
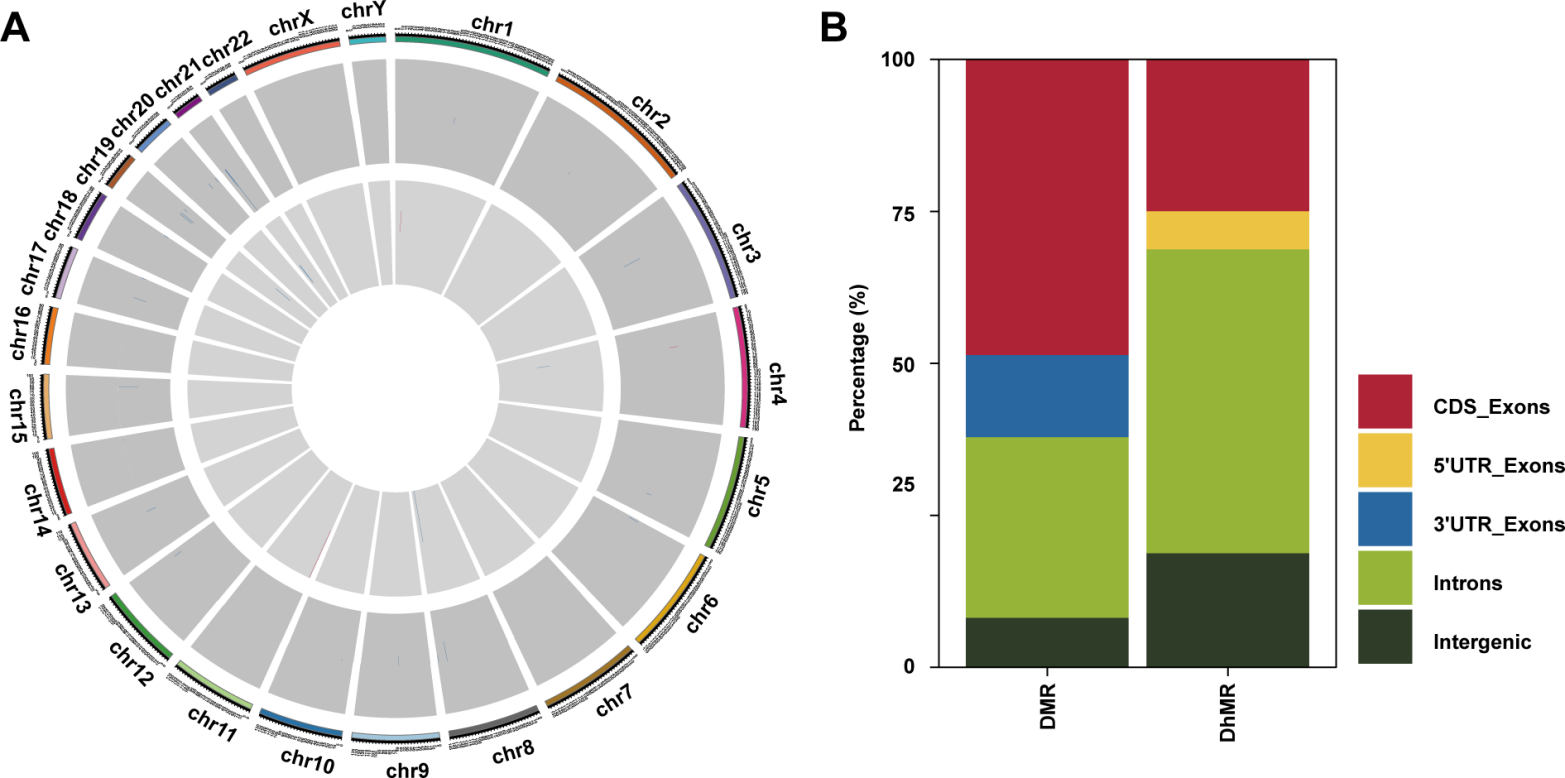
Identification of DMRs and DhMRs between *Cs*^+^ and *Cs*^-^ HCC tumors. **(A)** The Circos plot illustrates the genomic distribution of DMRs and DhMRs between *Cs*^+^ and *Cs*^-^ HCC tumors across all chromosomes. **(B)** Distribution of DMRs and DhMRs between *Cs*^+^ and *Cs*^-^ HCC tumors across genomic regions.

To further clarify the differences between *Cs*^+^ and *Cs*^-^ HCC tumors, we compared the methylated and hydroxymethylated regions between these tumor types to decipher the epigenetic mechanisms underlying their distinct responses to *Cs* infection. By comparing the 5mC and 5hmC profiles between the two groups, we identified 29 DMRs and 13 DhMRs, of which 1 DMR and 3 DhMRs were hyper-methylated/hyper-hydroxymethylated, while 28 DMRs and 10 DhMRs were hypomethylated/hypohydroxymethylated (**Supplementary Tables 8, 9**). Meanwhile, the location of the DMR and DhMRs in chromosome was exhibited (**Figure 5A**). Furthermore, DMRs were found to predominantly located in CDS exons and introns (**Figure 5B**). Although DhMRs show a similar distribution pattern, there was some difference in proportion.

Finally, we also analyzed the DMRs and DhMRs between the *Cs*^+^ and *Cs*^-^ HCC adjacent non-tumor tissues. A total of 24 DMRs and 11 DhMRs were detected by comparing the 5mC and 5hmC profiles between the two groups (**Supplementary Tables 10, 11**). The distribution of the DMRs and DhMRs in chromosomes and genomic regions was also displayed (**Supplementary Figures 7A, B**).

### 3.5 Integrative analysis of DNA methylation and gene expression in *Cs*^+^ and *Cs*^-^ HCC tumors

**Figure 6:**
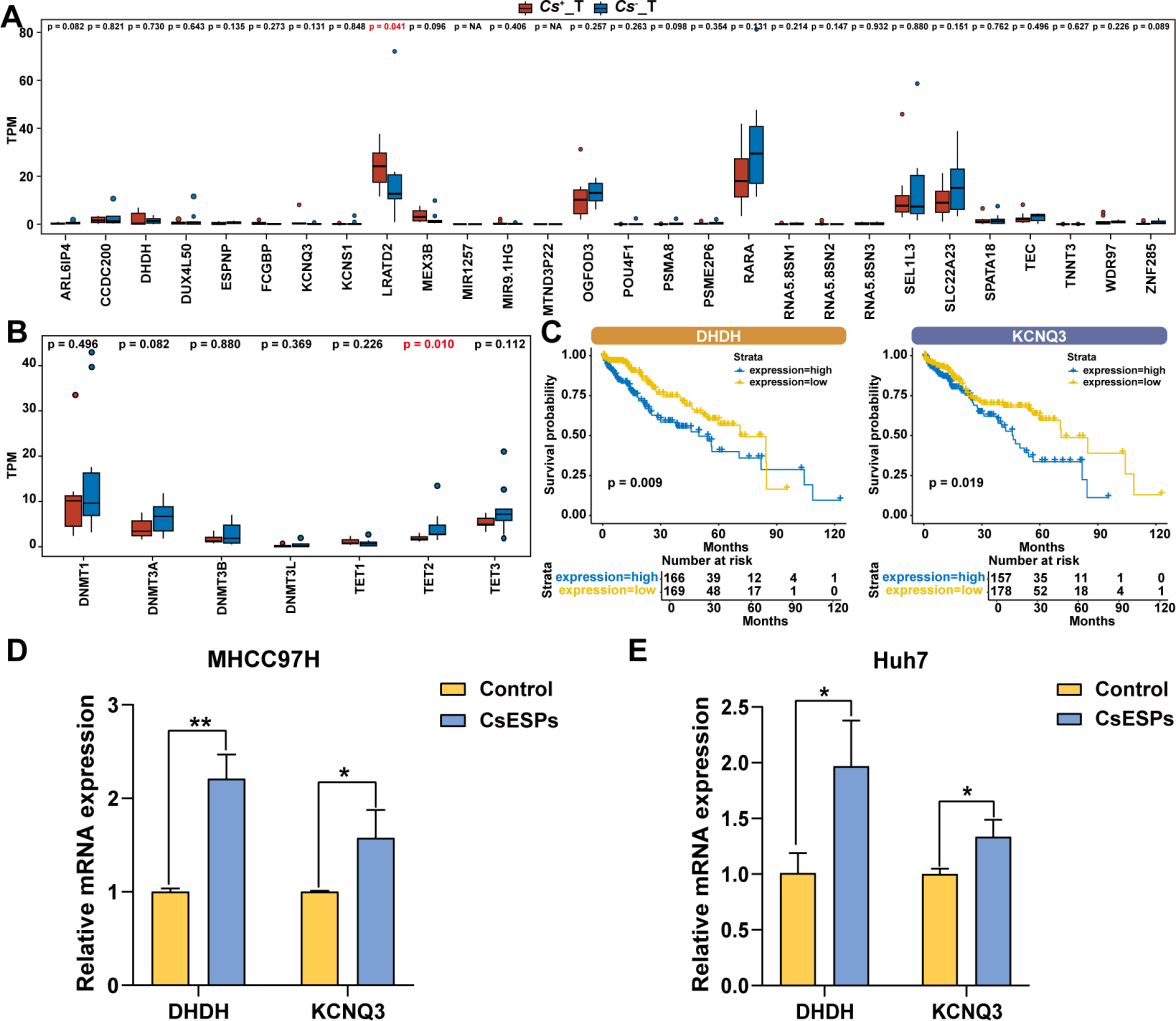
Integrated analysis of RNA-seq data from *Cs*^+^ and *Cs*^-^ HCC tumors. **(A)** Different expression level of DAGs between *Cs*^+^ and *Cs*^-^ HCC tumors. **(B)** Different expression levels of DNA methylation-related genes between *Cs*^+^ and *Cs*^-^ HCC tumors. **(C)** The Kaplan-Meier curves shows the association between the gene expression level of DAGs/DhAGs DHDH (*p* = 0.009) and KCNQ3 (*p* = 0.019) and survival outcomes in TCGA-LIHC cohort. D, **(E)** qRT-PCR validation of DHDH and KCNQ3 expression in MHCC97H **(D)** and Huh7 **(E)** cells. The symbol * indicates *p* < 0.05, and ** indicates *p* < 0.01.

To elucidate the impact of DNA methylation modifications on gene expression in HCC following *Cs* infection, we integrated RNA-seq data from *Cs*^+^ and *Cs*^-^ HCC tumors to analyze all differentially methylated/hydroxymethylated-associated genes (DAGs and DhAGs) expression levels between *Cs*^+^ and *Cs*^-^ HCC tumors (**Supplementary Tables 12, 13**). Notably, LRATD2, a gene associated with cell proliferation and cancer metastasis (Cheng et al., 2016; Huang et al., 2021; Jin et al., 2024), exhibited markedly higher expression in *Cs*^+^ HCC compared to *Cs*^-^ HCC (**Figure 6A**). What’s more, we further examined the expression of DNA methylation-related genes, focusing on their potential involvement in the epigenetic reprogramming observed in *Cs*^+^ HCC. Interestingly, the expression of TET2, a key enzyme involved in active DNA demethylation and 5hmC generation, was significantly downregulated in *Cs*^+^ HCC tumors relative to *Cs*^-^ HCC tumors (**Figure 6B**). Additionally, the survival outcomes of all DAGs and DhAGs in TCGA-LIHC cohort were also analyzed, and it revealed that high expression levels of DHDH and KCNQ3 were associated with significantly worse overall survival in HCC patients, which highlighted the potential prognostic value of DAGs/DhAGs expression and their critical role in HCC progression (**Figure 6C**). Moreover, the lack of significant association between the gene expression levels of DAGs/DhAGs and survival outcomes in the TCGA-LIHC cohort was also presented (**Supplementary Figure 8**). To further validate these findings, we treated HCC cell lines with CsESPs. qRT-PCR analysis demonstrated that CsESPs treatment significantly upregulated the expression of DHDH and KCNQ3 in both MHCC97H and Huh7 cells compared with PBS-treated controls (**Figures 6D, E**). Collectively, these findings indicate a potential association between parasite-related epigenetic alterations, transcriptional dysregulation, and clinical outcomes, highlighting DHDH and KCNQ3 as candidate mediators of *Cs*-related hepatocarcinogenesis.

### 3.6 Clinical significance of DMRs and DhMRs between *Cs*^+^ and *Cs*^-^ HCC tumors

**Figure 7:**
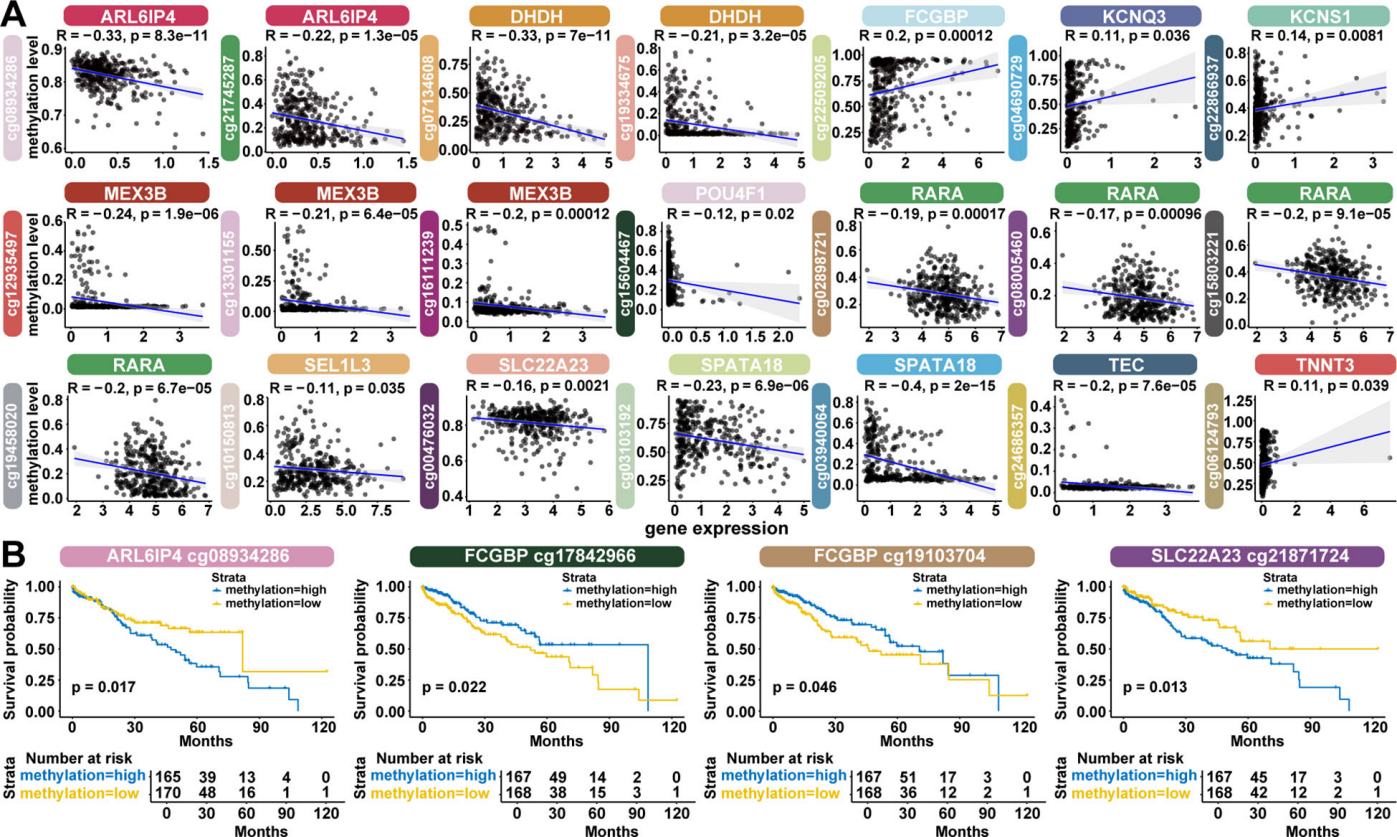
Correlation between DMRs/DhMRs caused by *Cs* infection and survival outcomes in the TCGA-LIHC cohort. **(A)** Spearman correlation was used to assess the relationship between methylation levels of DAGs/DhAGs-related promoter CpG sites and their gene expression levels between *Cs*^+^ and *Cs*^-^ HCC tumors. **(B)** The Kaplan-Meier curves shows the association between the methylation level of DAGs-related promoter CpG sites and survival outcomes.

To investigate the relationship between promoter DNA methylation and hydroxymethylation levels and gene expression, we analyzed the LIHC-TCGA dataset, integrating level 3 methylation data with paired RNA-seq profiles. We identified 21 promoter CpG sites associated with the methylation levels of DAGs/DhAGs that were significantly correlated with their respective gene expression levels in the TCGA-LIHC cohort (**Figure 7A**). Among these, 17 CpG sites demonstrated a negative correlation with gene expression, while 4 CpG sites exhibited a positive correlation. For instance, the promoter CpG sites cg08934286 of ARL6IP4 demonstrated a Spearman correlation coefficient of -0.33 (*p* = 8.3e-11), indicating that higher methylation at its promoter was associated with decreased gene expression. Next, we examined the impact of promoter methylation on overall survival in HCC patients. Kaplan-Meier survival analyses revealed that methylation levels at specific CpG sites within DAGs and DhAGs promoters were significantly associated with patient prognosis. For example, hypermethylation at the promoter CpG sites cg17842966 and cg19103704 of FCGBP was positively correlated with longer survival, while hypermethylation at the promoter CpG sites cg08934286 of ARL6IP4 and cg21871724 of SLC22A23 was associated with poorer survival outcomes (all log-rank *p* < 0.05) (**Figure 7B**). Last but not least, the lack of significant linear correlation between the methylation levels of certain DAGs-related promoter CpG sites and gene expression in the TCGA-LIHC cohort is shown in **Supplementary Figure 9**, and the lack of significant association between methylation levels of DAGs/DhAGs-related promoter CpG sites and survival outcomes is shown in **Supplementary Figure 10**.

## 4 Discussion

DNA methylation is one of the major causes of cancer progression and is associated with abnormal gene expression (Bhootra et al., 2023). Previous studies have shown a significant relationship between DNA methylation and the initiation, development, and metastasis of HCC (Nagaraju et al., 2022; Su et al., 2024). Interestingly, our study revealed that *Cs* infection remodeled the DNA methylation and hydroxymethylation patterns in HCC, which suggest *Cs*-infection HCC was a special subtype (**Figure 5**). Specifically, 29 DMRs and 13 DhMRs were identified in *Cs*^+^ HCC tumors compared to *Cs*^-^ HCC tumors, which would remodel the expression profile of genes (**Supplementary Tables 8, 9**). Previous studies have shown that *Cs* infection accelerates the malignant progression of HCC through various mechanisms, such as stimulating the proliferation of hepatic progenitor cells, activating the RAS/MAPK/ERK and PI3K/AKT signaling pathways, enhancing stem cell properties, and promoting angiogenesis (Wang C. et al., 2021; Qi et al., 2022; Lin et al., 2024; Wei et al., 2024). Significantly, our study is the first to highlight, from an epigenetic perspective, that the reprogramming of the genomic methylation landscape is also a crucial pathway in modulating *Cs*-infected HCC progression, which will expand our understanding of the specific characteristics of *Cs*-infected HCC.

In this study, 28 DMRs/DhMRs related genes were identified in *Cs*^+^ HCC, two genes (DHDH and KCNQ3) of which were significantly associated with the overall survival of HCC (**Figure 6C**). DHDH has been identified as a gene correlated with lenvatinib resistance and is implicated in the metabolic pathways related to liver cancer progression (Yang et al., 2021; Huang et al., 2022), and KCNQ3 activates the upstream signaling network of CAMK1, involved in regulating key biological processes such as cell proliferation, protein transport, has been implicated in HCC progression (Wang et al., 2014). Although DHDH and KCNQ3 did not show significant expression differences between *Cs*^+^ and *Cs*^−^ HCC tumors, their differential methylation and hydroxymethylation patterns suggest potential epigenetic modulation rather than direct transcriptional alteration. Such locus-specific methylation changes may influence gene activity in a context-dependent manner—for example, by affecting chromatin accessibility or enhancer–promoter interactions—without leading to substantial changes in bulk mRNA expression. Moreover, their strong prognostic associations in the TCGA cohort indicate that these genes may play broader roles in HCC progression, independent of *Cs* infection status. Collectively, these findings suggest that epigenetic remodeling of key regulatory genes, even in the absence of large expression shifts, may contribute to the biological heterogeneity and clinical outcomes of HCC. Moreover, we observed a reduction in the expression of TET2, which was a key DNA demethylase that converts 5mC to 5hmC, in *Cs*^+^ HCC tumors compared to *Cs*^-^ HCC tumors based on RNA-seq data (**Figure 6B**). The reduced expression of TET enzymes is often linked to decreased 5hmC levels (Bray et al., 2021; Zhang et al., 2023; López-Moyado et al., 2024). Previous studies have demonstrated that the loss of TET2 disrupts DNA methylation regulation, reducing 5hmC levels and altering methylation patterns, which in turn promotes HCC progression by modulating gene expression and facilitating tumor development (Wang et al., 2019; Guo et al., 2023). Therefore, our findings suggest that *Cs* infection may promote HCC carcinogenesis by inhibiting the TET2-mediated DNA demethylation pathway, leading to aberrant DNA methylation and hydroxymethylation patterns that drive tumorigenesis.

To further explore the clinical relevance of these epigenetic modifications, we also performed survival analyses of DAGs/DhAGs promoter CpG sites in *Cs*-infected HCC based on TCGA-LIHC data. Firstly, we identified 21 promoter CpG sites of DAGs/DhAGs, whose methylation levels were significantly associated with gene expression levels, with most showing a negative correlation (**Figure 7A**). These findings are consistent with the well-established principle that hypermethylation of promoter regions leads to gene silencing, while hypomethylation can activate gene expression (Russo et al., 2021). Survival analysis of these promoter CpG sites in the TCGA-LIHC database revealed four CpG sites whose methylation levels were significantly associated with patient survival (**Figure 7B**). Among them, the promoter CpG site cg08934286 also showed a strong negative correlation with the expression of its corresponding gene, ARL6IP4 (**Figure 7A**). Significantly, Bao et al. reported that ARL6IP4 was upregulated in HCC tumor tissues and was likely involved in cancer progression, contributing to the malignancy of HCC as part of a prognostic gene signature (Bao and Wu, 2024). In addition to ARL6IP4, we also observed altered methylation patterns of FCGBP and SLC22A23 in *Cs*^+^ HCC. FCGBP encodes an Fc fragment–binding glycoprotein that participates in mucosal immunity and epithelial barrier maintenance, and its aberrant methylation has been linked to chronic inflammation–associated tumorigenesis and immune evasion in liver and gastrointestinal cancers (Liu et al., 2022; Fu et al., 2025). SLC22A23, a solute carrier family member, has been implicated in metabolic reprogramming and drug transport in HCC, where promoter hypomethylation may enhance its expression and promote tumor cell survival and invasion (Serrano León et al., 2014; Arora et al., 2021). These findings suggest that *Cs* infection may contribute to HCC progression by reshaping the methylation status of genes involved in immune regulation and metabolic adaptation. It is worth noting that the correlations between promoter methylation and gene expression in these genes (R < 0.4) are statistically significant but relatively modest. This likely reflects the multifactorial nature of epigenetic regulation in HCC, where transcriptional output is jointly influenced by DNA methylation, histone modifications, chromatin accessibility, and transcription factor binding. Therefore, while promoter CpG methylation provides meaningful insight into epigenetic remodeling in *Cs*-related HCC, additional regulatory mechanisms are likely to coexist and warrant further investigation. Collectively, these results suggest that *Cs* infection alters the expression profile and methylation status of key DAGs/DhAGs, such as ARL6IP4, FCGBP, and SLC22A23, which may collectively contribute to the epigenetic reprogramming and unfavorable prognosis of HCC. In the present study, we observed that while a subset of promoter CpG sites showed significant correlations with gene expression and patient survival, a proportion did not (**Supplementary Figures 9, 10**), highlighting the functional heterogeneity of CpG sites and the complexity of epigenetic regulation in HCC (Wang et al., 2022). Gene expression is regulated by multiple mechanisms, including not only promoter DNA methylation but also enhancers, transcription factor binding, chromatin accessibility, and non-coding RNAs (Loaeza-Loaeza et al., 2020; Wang et al., 2026). Therefore, DNA methylation alone may not fully explain transcriptional variation. Moreover, not all promoter CpG sites contribute equally to transcriptional regulation. CpG sites located within key regulatory regions, particularly those proximal to transcription start sites, tend to exhibit stronger associations with gene expression, whereas others may display weaker or context-dependent effects depending on cell state or signaling conditions (Chi et al., 2025; Heery and Schaefer, 2025; Marei, 2025). Importantly, the relationship between DNA methylation and gene expression is not strictly linear, but instead depends on genomic context and environmental cues. DNA methylation can exert context-dependent regulatory effects, resulting in either positive or negative correlations with gene expression (Palczewski et al., 2025). One potential explanation for this complexity is the presence of 5hmC, which represents an intermediate and potentially independent epigenetic mark (Chera et al., 2024). The dynamic interconversion between 5mC and 5hmC may further contribute to nonlinear or even decoupled relationships between promoter methylation and transcriptional output (Chera et al., 2024; Palczewski et al., 2025).

Our analysis reveals a significant correlation between DAGs and their methylation sites with the prognosis of *Cs*-infected HCC, suggesting that these epigenetic modifications may play a critical role in the progression and poor prognosis of this disease. Given the importance of methylation in gene regulation, targeting these epigenetic changes could represent a promising therapeutic strategy. Notably, Clustered Regularly Interspaced Short Palindromic Repeats (CRISPR) technology can be used to screen and validate differentially expressed genes and their methylation sites related to HCC, as well as to develop targeted therapies by editing these genes and modifying their expression or methylation status for improved treatment outcomes (Wu et al., 2020; Yu et al., 2023). By combining dCas9 with activation or repression factors, as well as methyltransferases, CRISPR enables the precise regulation of gene expression and epigenetic modifications (Wang Y. et al., 2021). Currently, clinical trials using CRISPR/Cas9 for various cancers and genetic disorders have shown some promising results, laying the groundwork for the potential future application of CRISPR/Cas9-based tools in precision medicine (Chavez et al., 2023). In future studies, we plan to utilize CRISPR/dCas9-based approaches to perform targeted functional validation by selectively modifying methylation at promoter CpG sites cg17842966 and cg19103704 (FCGBP), cg08934286 (ARL6IP4), and cg21871724 (SLC22A23), as well as performing gene knockout or overexpression of key differential genes identified in this study, including DHDH, KCNQ3, TET2, and ARL6IP4, to investigate their effects on gene expression, tumor cell behavior, and potential therapeutic strategies for *Cs*-infected HCC.

In conclusion, our study firstly provides comprehensive genome-wide maps of 5mC and 5hmC at single-base resolution in *Cs*-infected and non-*Cs*-infected HCC. By integrating these epigenetic profiles with RNA-seq data and the TCGA database, we identified key differentially methylated sites and genes that are involved in *Cs*-associated HCC progression. These findings not only fill the gap in the 5mC and 5hmC maps associated with *Cs*-infected HCC but also highlight the potential of DAGs and their methylation sites for predicting prognosis and as therapeutic targets in HCC. However, the limited sample number of *Cs*-infected HCC cases may restrict statistical power and generalizability. Thus, these findings should be regarded with caution. Future studies should incorporate larger independent cohorts to further validate the clinical relevance of the identified biomarkers. In addition, further validation through *in vitro* and *in vivo* experiments, such as gene knockout or overexpression of methylation-related genes, is necessary to fully elucidate the epigenetic mechanisms underlying *Cs*-infected HCC and to evaluate the clinical significance of the identified biomarkers in larger cohorts. Spatially resolved transcriptomic approaches, including *in situ* RNA detection techniques (e.g., WISH), may also provide complementary insights into the tissue-specific expression patterns of key genes in *Cs*-infected lesions and may further contribute to the development of potential diagnostic strategies for HCC in the future.

## 5 Conclusions

This study investigates the epigenetic changes in *Cs*-infected HCC using whole-genome bisulfite sequencing and whole-genome oxidative bisulfite sequencing to map 5-methylcytosine and 5-hydroxymethylcytosine profiles at single-nucleotide resolution. We identified 29 differentially methylated regions and 13 differentially hydroxymethylated regions in *Cs*-infected HCC, which were associated with 28 DAGs/DhAGs. The methylation levels of four promoter CpG sites of DAGs/DhAGs were found to correlate with prognosis in *Cs*-infected HCC, while two key DMR/DhMR-associated genes, DHDH and KCNQ3, were significantly linked to overall survival of HCC. Our study provides the first detailed epigenetic map of *Cs*-associated HCC, uncovering novel insights into potential biomarkers and therapeutic targets that could enhance prognostic predictions and inform treatment strategies for patients affected by this condition.

## Data Availability

The datasets presented in this study can be found in online repositories. The names of the repository/repositories and accession number(s) can be found in the article/**Supplementary Material**.
