# Supplementary material for: Clonorchis sinensis infection alters the methylation and hydroxymethylation of hepatocellular carcinoma
Source: Front Cell Infect Microbiol. 2026 Jul 3;16:1799003. doi: 10.3389/fcimb.2026.1799003 (PMC13375505; doi:10.3389/fcimb.2026.1799003)
Supplement: Supplementary file 1 [file DataSheet1.pdf]

Supplementary Material

1.1 Supplementary Figures

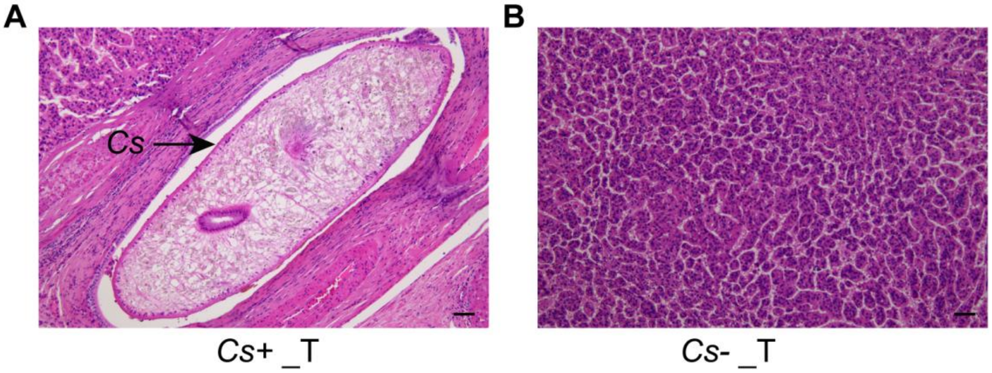

Fig. S1. Histological and parasitological characterization of *Cs*$^{+}$ and *Cs*$^{-}$ HCC tumor.

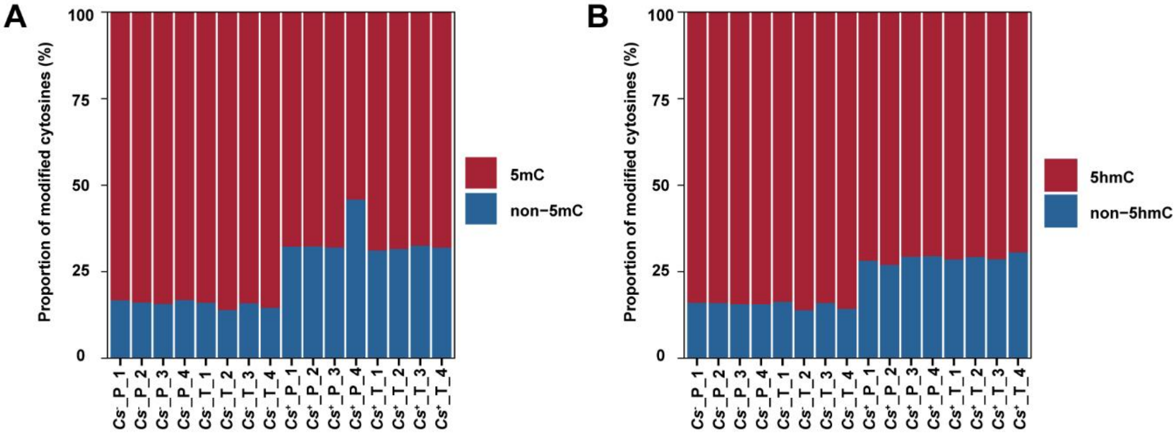

Fig. S2. Staking bar plot shows the proportion of 5mC (A) and 5hmC (B) at CpG sites in each sample.

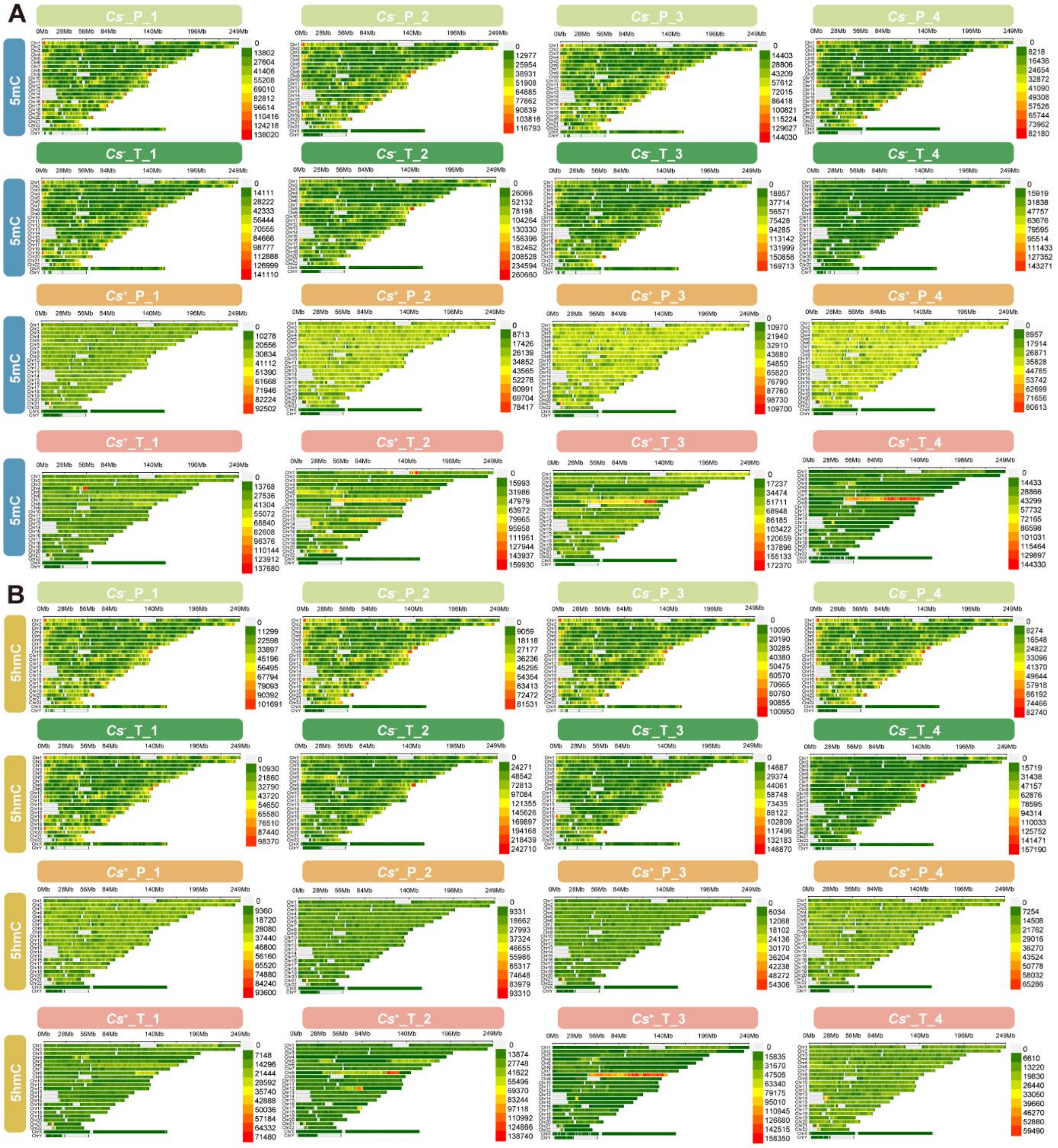

Fig. S3. The density distribution of methylation (A) and hydroxymethylation (B) sites on chromosomes in each sample.

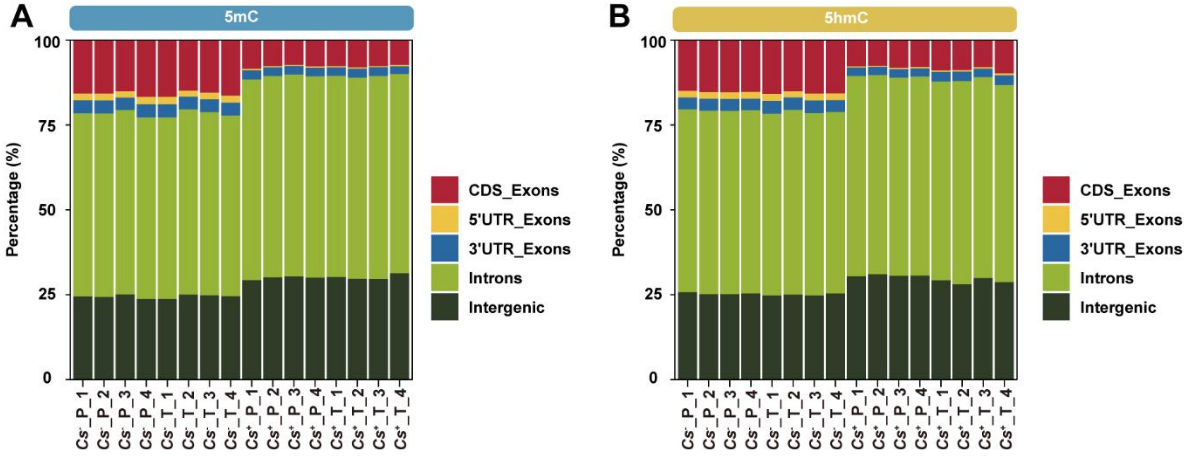

Fig. S4. Distribution of 5mC (A)and 5hmC (B) across functional genomic regions in each sample.

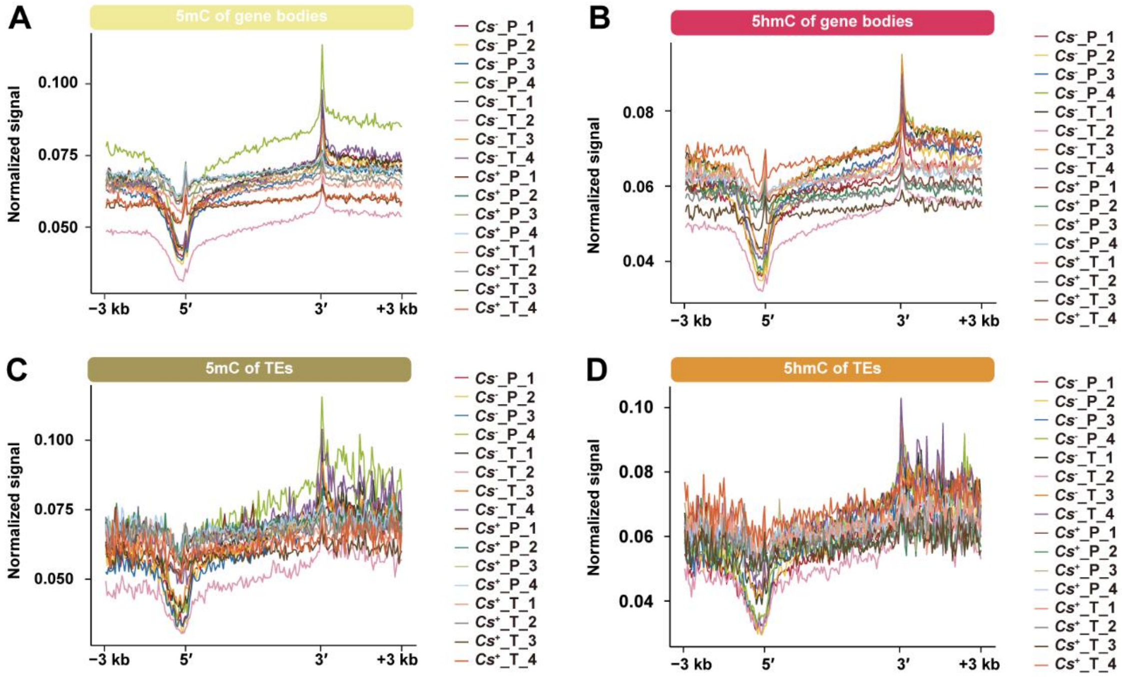

Fig. S5. Average DNA methylation and hydroxymethylation levels of gene bodies and TEs in each sample. A, B. Average DNA methylation **(A)** and hydroxymethylation **(B)** levels of gene bodies in *Cs*$^{+}$ HCC and *Cs*$^{-}$ HCC patients. **C, D.** Average DNA methylation **(C)** and hydroxymethylation **(D)** levels of TEs in *Cs*$^{+}$ and *Cs*$^{-}$ HCC patients.

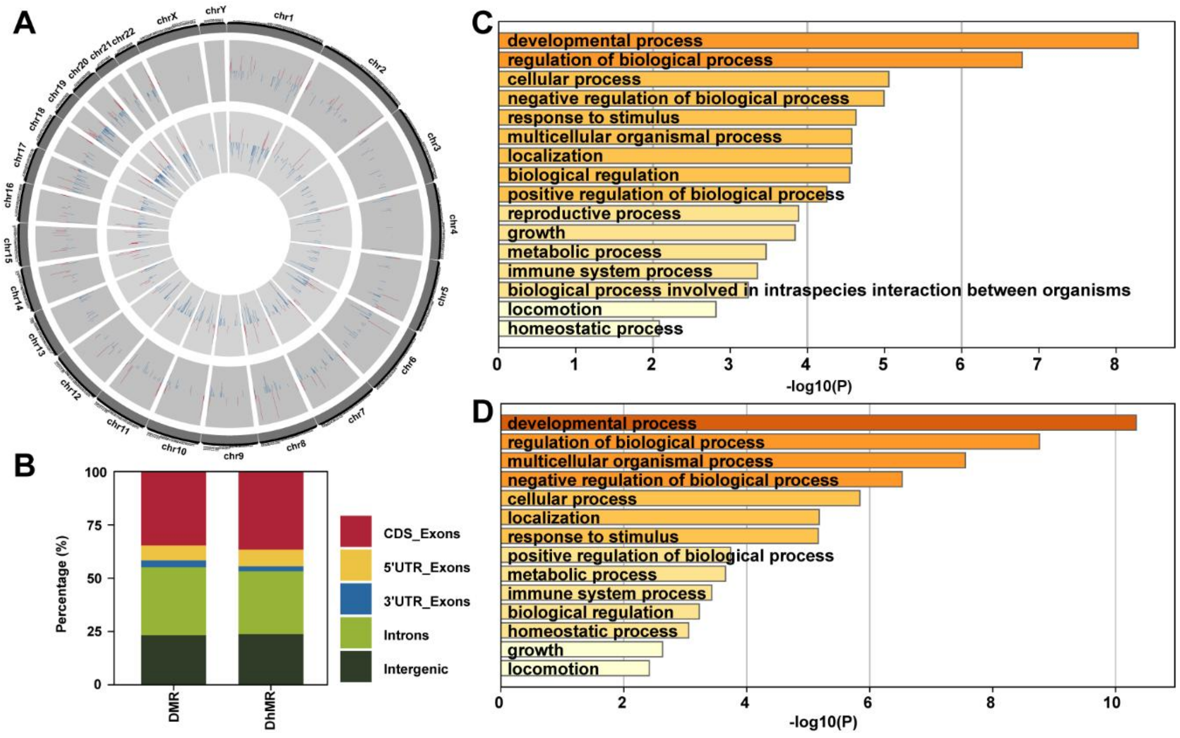

Fig. S6. Identification of DMRs and DhMRs between *Cs*$^-$ HCC tumors and adjacent non-tumor tissues. A. The Circos plot illustrates the genomic distribution of DMRs and DhMRs between *Cs*$^-$ HCC tumors and adjacent non-tumor tissues across all chromosomes. **B.** Distribution of DMRs and DhMRs between *Cs*$^-$ HCC tumors and adjacent non-tumor tissues across genomic regions. **C, D.** GO analysis of DAGs/DhAGs based on DMRs (**C**) and DhMRs (**D**) between *Cs*$^-$ HCC tumors and adjacent non-tumor tissues.

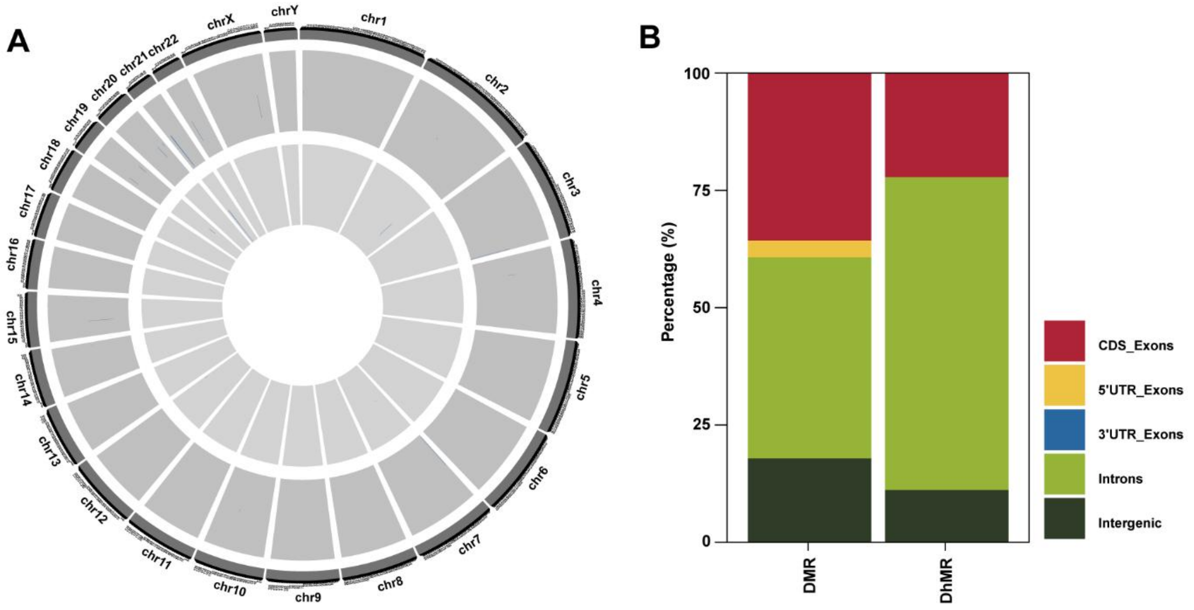

Fig. S7. Identification of DMRs and DhMRs between *Cs*$^{+}$ and *Cs*$^{-}$ HCC adjacent non-tumor tissues. A. The Circos plot illustrates the genomic distribution of DMRs and DhMRs between *Cs*$^{+}$ and *Cs*$^{-}$ HCC adjacent non-tumor tissues across all chromosomes. **B.** Distribution of DMRs and DhMRs between adjacent non-tumor tissues across genomic regions.

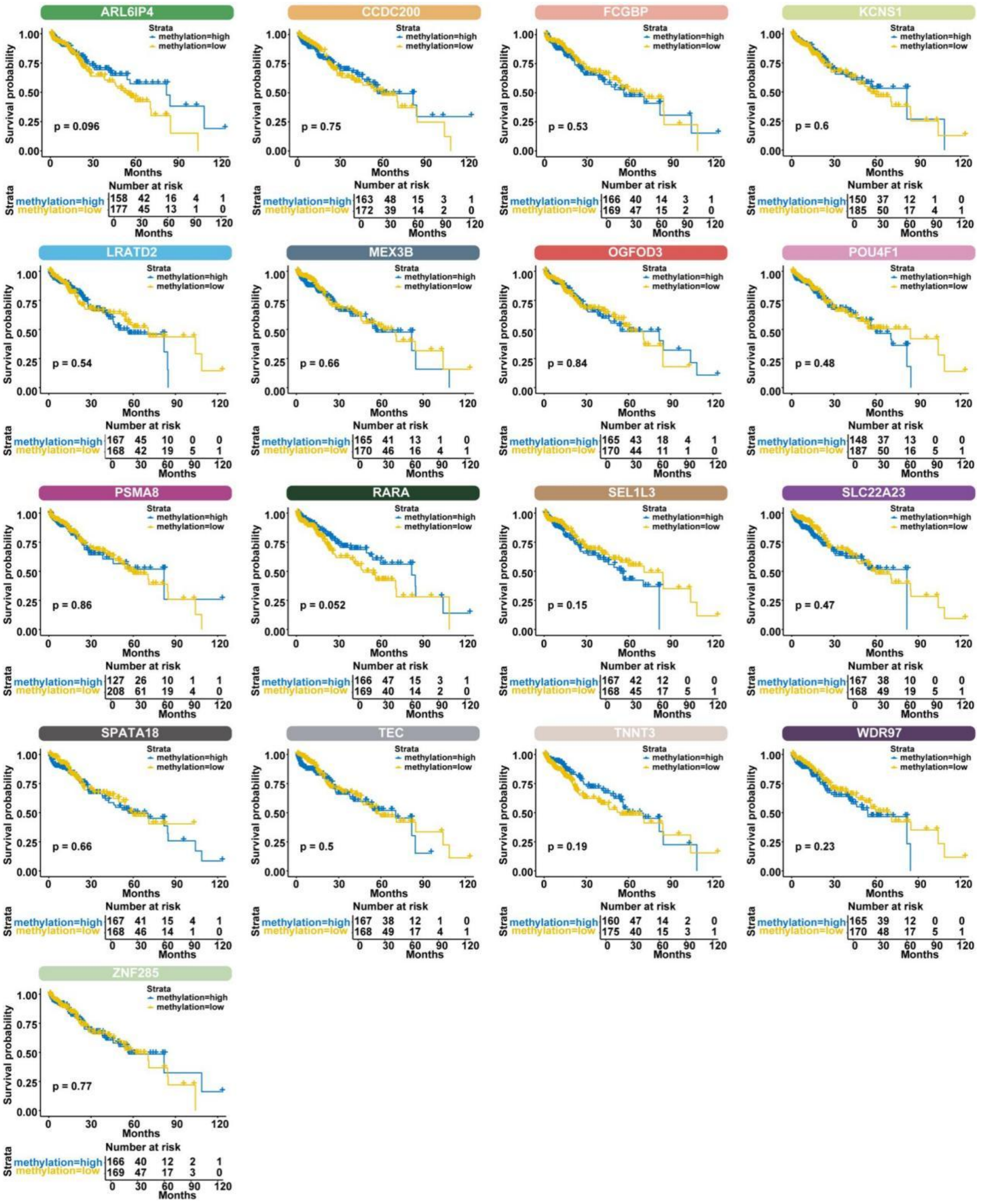

Fig. S8. The Kaplan-Meier curves show the lack of significant association between the gene expression level of DAGs/DhAGs and survival outcomes in TCGA-LIHC cohort.

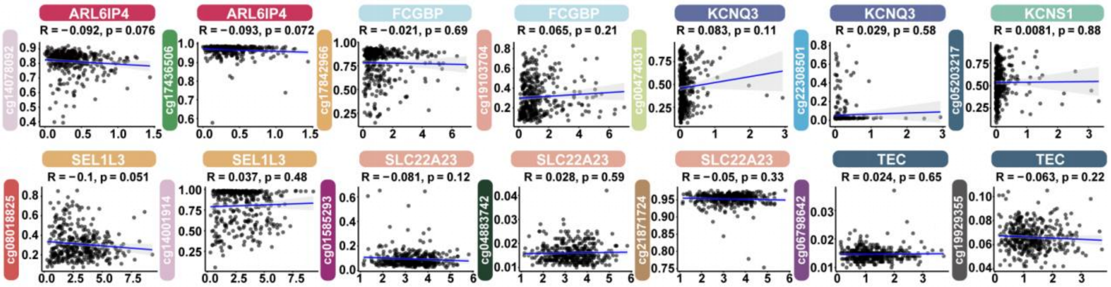

Fig. S9. The linear plot shows the lack of significant linear correlation between the methylation levels of certain DAGs-related promoter CpG sites and gene expression levels in the TCGA-LIHC cohort.

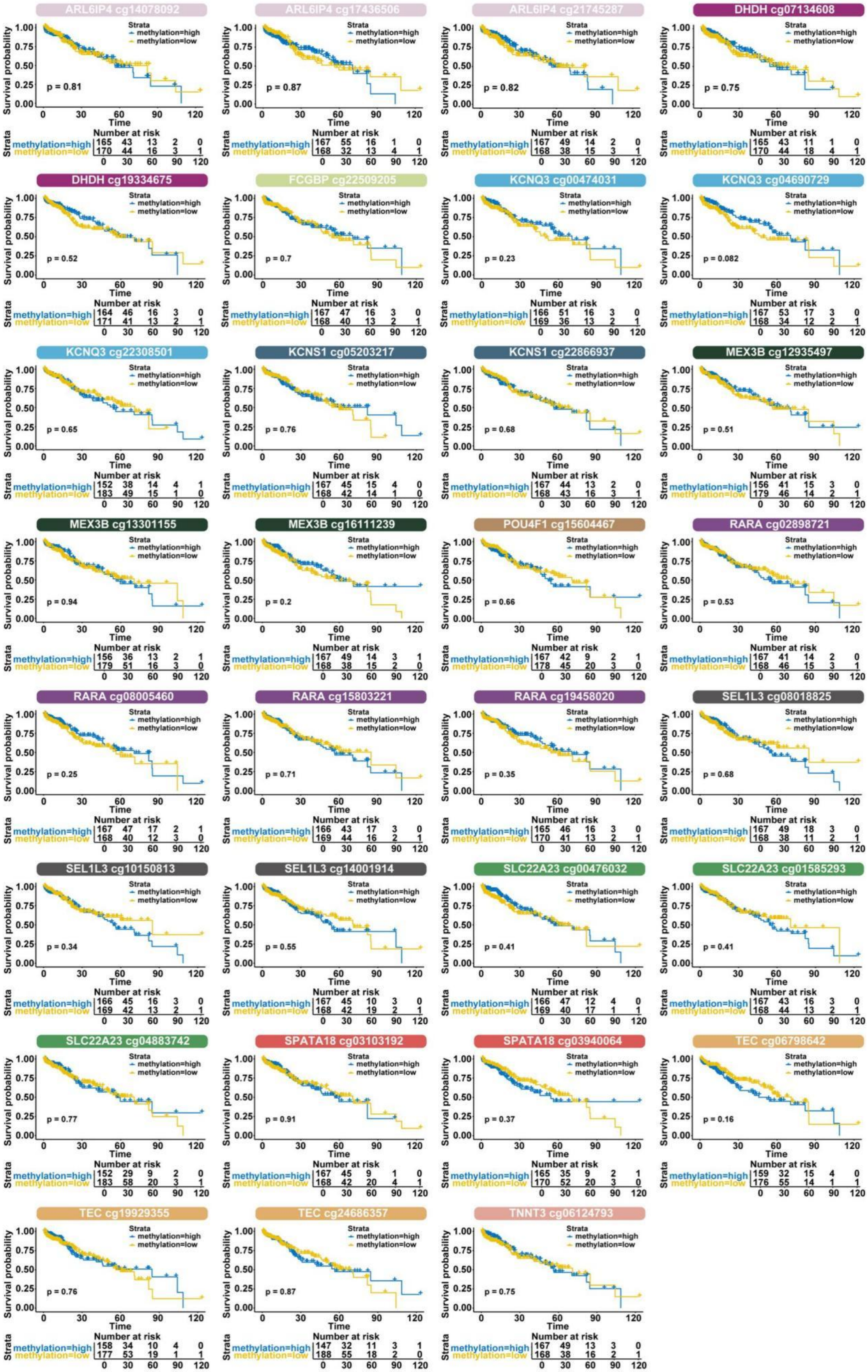
Survival probability
Time
Strata
methylation=high
methylation=low
Number at risk
ARL6IP4 cg14078092
p = 0.81
methylation=high 165 43 13 2 0
methylation=low 170 44 16 3 1
ARL6IP4 cg17436506
p = 0.87
methylation=high 167 55 16 1 0
methylation=low 168 32 13 4 1
ARL6IP4 cg21745287
p = 0.82
methylation=high 167 49 14 2 0
methylation=low 168 38 15 3 1
DHDH cg07134608
p = 0.75
methylation=high 165 43 11 1 0
methylation=low 170 44 18 4 1
DHDH cg19334675
p = 0.52
methylation=high 164 46 16 3 0
methylation=low 171 41 13 2 1
FCGBP cg22509205
p = 0.7
methylation=high 167 47 16 3 0
methylation=low 168 40 13 2 1
KCNQ3 cg00474031
p = 0.23
methylation=high 166 51 16 3 0
methylation=low 169 36 13 2 1
KCNQ3 cg04690729
p = 0.082
methylation=high 167 53 17 3 0
methylation=low 168 34 12 2 1
KCNQ3 cg22308501
p = 0.65
methylation=high 152 38 14 4 1
methylation=low 183 49 15 1 0
KCNS1 cg05203217
p = 0.76
methylation=high 167 45 15 4 0
methylation=low 168 42 14 1 0
KCNS1 cg22866937
p = 0.68
methylation=high 167 44 13 2 0
methylation=low 168 43 16 3 1
MEX3B cg12935497
p = 0.51
methylation=high 156 41 15 3 0
methylation=low 179 46 14 2 1
MEX3B cg13301155
p = 0.94
methylation=high 156 36 13 2 1
methylation=low 179 51 16 3 0
MEX3B cg16111239
p = 0.2
methylation=high 167 49 14 3 1
methylation=low 168 38 15 2 0
POU4F1 cg15604467
p = 0.66
methylation=high 167 42 9 2 1
methylation=low 178 45 20 3 0
RARA cg02898721
p = 0.53
methylation=high 167 41 14 2 0
methylation=low 168 46 15 3 1
RARA cg08005460
p = 0.25
methylation=high 167 47 17 2 1
methylation=low 168 40 12 3 0
RARA cg15803221
p = 0.71
methylation=high 166 43 17 3 0
methylation=low 169 44 16 2 1
RARA cg19458020
p = 0.35
methylation=high 165 46 16 3 0
methylation=low 170 41 13 2 1
SEL1L3 cg08018825
p = 0.68
methylation=high 167 49 18 3 0
methylation=low 168 38 11 2 1
SEL1L3 cg10150813
p = 0.34
methylation=high 166 45 16 3 0
methylation=low 169 42 13 2 1
SEL1L3 cg14001914
p = 0.55
methylation=high 167 45 10 3 0
methylation=low 168 42 19 2 1
SLC22A23 cg00476032
p = 0.41
methylation=high 166 47 12 4 0
methylation=low 169 40 17 1 1
SLC22A23 cg01585293
p = 0.41
methylation=high 167 43 16 3 0
methylation=low 168 44 13 2 1
SLC22A23 cg04883742
p = 0.77
methylation=high 152 29 9 2 0
methylation=low 183 58 20 3 1
SPATA18 cg03103192
p = 0.91
methylation=high 167 45 9 1 0
methylation=low 168 42 20 4 1
SPATA18 cg03940064
p = 0.37
methylation=high 165 35 9 2 1
methylation=low 170 52 20 3 0
TEC cg06798642
p = 0.16
methylation=high 159 32 15 4 0
methylation=low 176 55 14 1 1
TEC cg19929355
p = 0.76
methylation=high 158 34 10 4 0
methylation=low 177 53 19 1 1
TEC cg24686357
p = 0.87
methylation=high 147 32 11 3 1
methylation=low 188 55 18 2 0
TNNT3 cg06124793
p = 0.75
methylation=high 167 49 13 3 0
methylation=low 168 38 16 2 1

Fig. S10. The Kaplan-Meier curves shows the lack of significant association between the methylation levels of certain DAGs/DhAGs-related promoter CpG sites and survival outcomes in the TCGA-LIHC cohort.
